# Identification of aroma compounds in a commonly prescribed oral nutritional supplement and associated changes in olfactory abilities with human ageing

**DOI:** 10.1038/s41598-021-95915-6

**Published:** 2021-08-13

**Authors:** Sophie Lester, Leonardo Cornacchia, Camille Corbier, Moira A. Taylor, Charfedinne Ayed, Ni Yang, Mui Lim, Rob Linforth, Ian Fisk

**Affiliations:** 1 Division of Food Nutrition and Dietetics, School of Biosciences, University of Nottingham, Nottingham, UK; 2 Division of Physiology, Pharmacology and Neuroscience, School of Life Sciences, National Institute for Health Research (NIHR) Nottingham Biomedical Research Centre, University of Nottingham, Nottingham, UK; 3 Danone Nutricia Research, Uppsalalaan 12, 3584 CT Utrecht, The Netherlands; 4 The University of Adelaide, North Terrace, Adelaide, SA Australia

**Keywords:** Malnutrition, Geriatrics

## Abstract

Undernutrition is prevalent in the older adult population. Oral nutritional supplements (ONS) are a clinically effective nutritional intervention, however, patient acceptance of ONS can be limited by their palatability. While sensory attributes such as sweetness and mouthfeel have been investigated, the contribution made by aroma to the perceived flavour of ONS has not been studied. Firstly, this research aimed to identify the aroma active compounds within a commonly prescribed ONS using estimated odour activity values (OAV) and gas chromatography olfactometry mass spectrometry (GC-O-MS). Secondly, age related differences in olfactory detection were explored. Eight aroma active compounds were identified within the ONS, including diacetyl (sweet), isoamyl acetate (banana), dimethyl trisulfide (sulfur) and methanethiol (sulfur). When compared with younger adults (n = 24, 18–44 years), older adults (n = 24, 62–80 years) had higher detection thresholds for all aroma compounds and this was significant for isoamyl acetate (sweet, fruity) and methanethiol (sulfur) (p = 0.01 and p = 0.03, respectively). Thus, a decline in olfactory sensitivity was present in the older subjects included in the study, and this reduced detection sensitivity was aroma specific. Thus, older adults’ flavour perception of ONS likely depends on the combined effect of product factors (the aroma profile) along with age related consumer factors (the degree of impairment in perception). This is a fundamental study which will aid future research into how the aroma profile, and associated age related impairments in perception, shape the global perception of ONS for nutritionally at risk older individuals.

## Introduction

Undernutrition is commonly experienced in the older population^1^ and the aetiology includes a multitude of age related physiological, social and environmental factors, encompassed under the common term “The Anorexia of Ageing”^2^. The consequences include many comorbidities and adverse outcomes including disability and poor quality of life^2,3^ and results in major healthcare costs^1^. Undernutrition is estimated to cost at least £23.5 billion in the UK; with older adults accounting for 52% of this cost^1^. Considering this, it is critical to identify effective nutritional interventions to prevent and treat undernutrition in the older population.

Foods for special medical purposes, such as oral nutritional supplements (ONS), are prescribed to supplement, or replace the diet of individuals who are undernourished, or at risk of undernutrition. Most prescribed ONS are dairy based, protein and energy dense drinks, which are ready to drink, and as such they are easy to consume by individuals with poor mobility, cognition and/or dentition. Liquids are less satiating than nutritionally equivalent solids^4^ facilitating consumption of a larger quantity before termination of intake. The clinical effectiveness of ONS, when consumed, is well established^1^. Stratton et al.,^5^ conducted a ‘review of reviews’ and found that ONS consistently increased total nutritional intake concurrently with a significant overall reduction in mortality and a reduction in complications (such as infections and pressure ulcers) across patient groups.

In order to gain the clinical benefits associated with ONS, sufficient long term intake is essential. After ‘nutritional value’, Dhuibhir et al.,^6^ found that ‘patient palatability’ was the most important factors affecting ONS prescription in clinical practice. However, ONS are poorly tolerated by patients^7^ and many may not consume their full prescription; Gosney^8^ found average adherence to a prescribed course of ONS on an elder care ward was as low as 37% (average quantity consumed as a proportion of the quantity provided). Gosney^8^ found that the greatest waste was seen in those patients who disliked the taste of ONS and poor palatability has been proposed as a key factor limiting sufficient intake^9–12^.

ONS typically contain adequate levels of nutritionally essential macronutrients (carbohydrates, protein, and fat) and micronutrients (vitamins and minerals) to produce a nutritionally complete product. However, the composition of ONS plays a crucial role in ensuring palatability^13,14^. For example, the type and quantity of carbohydrate chosen in an ONS formulation (usually a simple sugar which are easy to digest and absorb^13^) must ensure sufficient quantities of glucose and calories for the patient, but the sweetness level must be considered; an overly sweet taste is undesirable for patients^8^. The protein choice is also crucial to ensure the palatability of the final product. For example, whey proteins (a high quality protein source) elicit a ‘mouth drying’ phenomenon during consumption, which can build up in intensity over multiple intakes or sips^15^ and becomes more intense with heat treatment of the protein ingredients^16^. During manufacture, ONS are typically heat treated to ensure adequate shelf life, but this treatment likely affects the sensory properties and palatability of the final product, including mouthfeel but also the generation of new flavours and aromas^13^. Some micronutrients have also been proposed as important in the generation of taste sensations in nutritional supplements^17^.

Olfaction is a sensory modality with great importance in food flavour because it is olfaction, which combined with the perception of taste during food consumption, creates the overarching perception of flavour. To the authors knowledge, no research has yet investigated the contribution made by volatile aroma compounds to the flavour and palatability of a commercial ONS. However, heat generated Maillard reactions products with undesirable sensory properties have been identified in heat treated dairy products, such as pungent, sulfur containing aroma compounds with low detection thresholds^18–22^. Uncovering the aroma which are present in a commonly prescribed dairy based ONS is a fundamental first area of investigation.

In addition to factors inherent to the product, older consumers experience unavoidable age related changes in their oro-sensory physiology which are likely to alter the sensory experience. It is well known that gustatory sensitivity declines with age, however, the extent and significance of this decline varies between taste modalities, tastants and studies^23^. Olfactory sensitivity declines considerably with age too, though, only a limited number of studies have investigated how ageing affects olfactory sensitivity to single aroma compounds^24–26^. Impairments in olfactory abilities likely involve age related alterations within the nose, olfactory epithelium, bulb, and higher brain structures^27^. In addition, it should also be noted that reduction in olfactory sensitivity is associated with early stages of some neurodegenerative diseases, for example Alzheimer’s disease and sporadic Parkinson’s disease^27^. Considering these age related changes, it is understandable how the palatability of foods, including ONS, may change over the life course.

Considering the importance of aroma compounds in flavour perception, this study firstly aimed to characterise the volatile profile of a commercial ONS and estimated Odour Activity Values (OAV) aimed to predict the volatiles with the potentially highest aroma activity. Gas chromatography olfactometry mass spectrometry (GC-O-MS) used human participants to validate these findings and confirm the aroma active volatiles which contribute most greatly to the flavour of ONS. Lastly, detection threshold tests were used to investigate the association between ageing and perceptual sensitivity to aroma active compounds.

## Materials and methods

This study was approved by the School of Bioscience Ethics Committee at the University of Nottingham (SBREC160140A). All experiments were performed in accordance with relevant guidelines and regulations.

### Gas chromatography-mass spectrometry (GC-MS)

#### Sample preparation

Commercial samples of a banana flavoured milk based oral nutritional supplement (ONS) were obtained from Danone Nutricia Research (Utrecht, The Netherlands) and stored at ambient temperature until analysis. Banana flavoured products were chosen as they are one of the most commonly prescribed flavours in the UK. For each sample, 7 mL of the ONS was placed into an opaque vial and 15 μl of a freshly prepared internal standard (0.1% 3-heptanone in ultra pure water) was added. The samples were analysed in triplicate.

#### GC-MS parameters

A trace 1300 series Gas Chromatograph coupled with a Single Quadrupole Mass Spectrometer (Thermo Fisher Scientific) was used. The sample was incubated at 37 °C for 15 min with shaking. Solid phase microextraction (SPME) was used to extract volatile aroma compounds from the sample headspace (extraction for 15 min, desorption for 1 min). The fibre used was a 50/30 μm DVB/CAR/PDMS SPME Fibre (Supelco, Sigma Aldrich, UK). The separation was carried out on a ZB WAX Capillary GC column (30 m × 0.25 mm × 1 μm). The column temperature was initially held at 50 °C for 1 min and then increased by 8 °C/min until the temperature reached 190 °C. The temperature was then increased by 15 °C/min until 250 °C was reached and then held for 0.5 min. Helium was used as a carrier gas and spitless mode was used at a constant carrier pressure of 30 psi. Full scan mode was used to detect volatile compounds (*m/z* 35 to 300). Volatile compounds were tentatively identified by comparison of each mass spectrum with spectra in reference libraries (NIST/SPA/NIH Mass Spectral Library, version 2.0, Faircom Corporation, U.S.) and by comparing calculated Linear Retention Indices (LRI) with those from either the literature or an internal database generated using authentic standards.

The relative abundance of each volatile compound present in the headspace was calculated by comparing the GC peak area to the peak area of the IS (of known abundance).

Odour activity values (OAV) give an indication of the relative contribution made by each aroma to the overall flavour and were calculated by dividing the volatile abundance by the odour thresholds (OT).

### Gas chromatography-olfactometry-mass spectrometry (GC-O-MS)

#### Participant recruitment

Six young adults (24–38 years, 4 male 2 female) and six older adults (67–81 years, 3 male 3 female) were recruited from Sutton Bonington Campus, University of Nottingham and local villages via posters and flyers. Interested participants were informed they were being recruited for a “smelling study” and must fit the criteria of being in good general health (healthy BMI, absence of physical or mental illness) and no known anosmia unrelated to ageing. Due to the high occurrence of medication use in older age, and to maintain ecological validity, medication use was not a criterion for exclusion, but the details of medication use were recorded (see Supplementary Table S2). All participants were new to sensory and flavour analysis.

#### Participant screening and training

Written informed consent was obtained and, to ensure they met the recruitment criteria, participants were asked to complete a questionnaire obtaining health, lifestyle and demographic information. In a training session (1 h), the 12 participants were familiarised with the GC-O-MS procedure and given the opportunity to practice smelling using the GC-O-MS equipment with a model blend of aroma compounds.

#### Sample preparation

Sample preparation was exactly as stated in the GC–MS analysis apart from the exclusion of the internal standard.

#### GC-O-MS parameters

The volatile compounds passing through the column were split in a 1:1 ratio, part was directed to the Mass Spectrometer and part was directed out of the oven via a transfer line (200 ºC), and exited at a glass sniffing port.

GC-O-MS analysis took place over 23 min. Data were collected by a combination of detection frequency (DF) (number of participants that detect the aroma) and posterior intensity rating (I) (participant rating of aroma intensity). The intensity rating used a simple 1–3 scale, which has been shown to be effective in GC-O-MS analysis^28^. Participants were asked to breathe normally through their nose in the sniffing port and state (i) exactly when they smelt an aroma (ii) give the intensity of the aroma (scale of detection: 1 = weak, 2 = intermediate, 3 = strong) (iii) give a description of the aroma. Participants were allowed to describe the aroma using their own terminology. To limit disruptions during smelling, participants verbally stated their ratings and descriptions, and these were recorded electronically by the experimenter. The detection time was used to identify odiferous regions of the chromatogram.

### Detection threshold tests

The threshold testing methods were based on the ATSM^29^ E679 3-alternative forced choice (3-AFC) method of limits. This method is suited for “best estimate” thresholds of populations or groups, which naturally include the large variation in threshold values. In addition, this method was especially suitable for naïve groups, such as untrained older adults, due to its relative simplicity^30^.

Oral nutritional supplements are a complex matrix of macronutrients and micronutrients. Thus, ONS can be a challenging matrix to reproduce, particularly whilst ensuring consistent aroma between samples or batches. Therefore, in line with existing literature values^31^, this initial study opted to use water as the aroma dilution medium.

Four aroma compounds were chosen from those detected during GC-O-MS (“Gas chromatography-olfactometry-mass spectrometry (GC-O-MS)”) to represent a variety of hedonic, chemical and sensory characteristics (sensory descriptors taken from Good Scents Company^32^. The aroma compounds and concentration ranges chosen were: isoamyl acetate (sweet, fruity) (25–6000 ppb), diacetyl (sweet, buttery) (1–200 ppb), methanethiol (cabbage, garlic) (0.025—6 ppb), dimethyl trisulfide (sulfurous, onion) (0.002—0.5 ppb). Suitable concentration ranges were determined by pilot threshold studies, with both age groups, leading up to the experiment. A total of six ascending concentration steps were chosen for each aroma and to encompass large variances in threshold values, but limit the number of samples, concentrations steps were increased by a factor of 3.

#### Materials

Three of the aroma compounds (isoamyl acetate (> 95%), 2,3-butandione (diacetyl) (97%) and dimethyl trisulfide (> 98%)) were purchased from Sigma Aldrich, U.S., and methanethiol (methyl mercaptan) (1 mg/mL in H_2_O) was purchased from Fisher Scientific UK Ltd.

##### Sample preparation

Aroma concentrations were prepared in ultrapure water using the serial dilution technique and blanks were water only. To prevent the loss of volatiles, dilutions were prepared no more than 24 h before the study day and stored in 500 mL Duran bottles, without headspace, and refrigerated until the day of the study session. On the day of a study session, for each sample, 5 mL of each dilution was pipetted into 28 mL glass screw top bottles and labelled with a randomised 3 digit code.

#### Participants

Using the same recruitment procedures and criteria the GC-O-MS analysis, 24 healthy young adults (18–44 years, 8 Male 16 Female) and 24 healthy older adults (62–80 years, 9 Male 15 Female) were recruited to take part.

#### Threshold screening and training session

Interested participants were invited to a group screening and training session where they were informed about the purpose and nature of the study. Participants provided informed consent before completing a health, lifestyle and demographic questionnaire.

In order to familiarise participants with the sensory procedure, participants were invited to practice the 3-AFC technique, for each aroma compound, by identifying odour containing bottle from the two blanks. The aroma compounds were presented at the next highest theoretical concentration step in the threshold range (concentration step seven). Participants were trained to smell the bottles in a standardised way: smell the bottles from left to right, shake the bottle and count to five before removing the lid, hold the bottle close to the nose without touching.

During this practice session, a small number of participants were unable to detect the odour containing bottle at the higher concentration (isoamyl acetate: 1 younger, diacetyl: 1 younger 1 older, methanethiol: 2 younger, dimethyl trisulfie: 2 younger, 2 older). These participants were possibly anosmic to the aroma (aroma-specific anosmia) and subsequently excluded from testing for that particular aroma in the threshold testing. Additionally, during the threshold testing, two older participants experienced acute respiratory illness which affected their normal smelling abilities. Subsequently, they were not included in the section they were scheduled to attend. To see final group sizes for each threshold test, see Supplementary Fig. S1.

#### Aroma compound pleasantness

As an additional measure, during the 3-AFC practice session, participants who correctly identified the odour containing bottle were asked to rate the pleasantness of each aroma compound using a 3 point categorical scale: Pleasant, Neutral, or Unpleasant, a recommended scale for hedonic testing with older adults due to the ease of use of category scales^30^.

#### Threshold testing procedure

All threshold study sessions took place in sensory booths designed to ISO Standards (ISO8589:1988) within The Sensory Science Centre (Sutton Bonington Campus, University of Nottingham). There were two study sessions per day (morning or afternoon) and sessions were mixed with both older and younger participants. Participants completed 2 different aromas per session with a compulsory 15 min break between different aroma compounds. The order that the different aroma compounds were presented was randomised and sample presentation order (1 aroma, 2 blanks) was randomised using Compusense Cloud (Compusense, Ontario, Canada).

Participants were presented with the 3 samples and asked one question “Which one sample smells different to the other two?” Between each set of 3 samples, participants were given a compulsory 1 min break and asked to neutralise their nose using a damp cloth. Answers were recorded electronically using Compusense Cloud (Compusense, Ontario, Canada).

### Statistical analysis

All statistical analysis was carried out using the software XLSTAT statistical and data analysis solution (version 20.6.01, Addinsoft, Long Island, NY, USA) or GraphPad Prism software (version 7.0, San Diego, CA, USA).

#### Gas chromatography-olfactometry-mass spectrometry

Detection frequency (DF) and posterior intensity ratings (I) were combined into a single value using the modified frequency (MF) method proposed by Dravnieks^33^ and used effectively in recent GC-O-MS studies^34–37^. MF can be calculated using the following formula:

$$MF \left(\%\right)=\sqrt{DF\left(\%\right)I\left(\%\right)}$$

whereby DF (%) is the total detection frequency, expressed as a percentage of total participants, and I (%) is the total intensity rating, expressed as a percentage of the maximum possible sum of intensity ratings. MF was calculated both for individual age groups and for data pooled from both groups. To control for noise a MF 50% cut off was used within each age group^34^.

#### Threshold tests and pleasantness ratings

Differences in aroma compound pleasantness ratings, between the older and younger groups, were analysed by Chi-Squared (X^2^) test at a significance level of p < 0.05.

Individual best estimate thresholds (BET) were determined by taking the geometric mean of each individual’s highest incorrect concentration and the correct concentration above this. If an individual’s threshold was above or below the highest possible, or lowest possible concentration, the next theoretical concentration step was used. Age group BET were determined by taking the geometric mean of all individual BET within an age group. Due to the data not being equally distributed, Mann–Whitney U test was used to compare threshold values between age groups, at a significance level of p < 0.05.

## Results and discussion

### Experiment 1: Identification of aroma active compounds in ONS

#### Gas chromatography-mass spectrometry (GC-MS)

GC–MS detected twenty nine volatile compounds, with wide ranging chemical and sensory properties in the headspace of the ONS (See Table 1). The main functional groups were: esters (8), aldehydes (6), sulfur-containing (4), furans (4), ketones (3), alcohols (2), phenylpropenes (1) and monoterpenes (1).

**Table 1:** Volatile compounds detected in the headspace of the ONS beverage, ordered by functional group and relative abundance.

LRI	Literature LRI^A^	Volatile compound name	m/z	CAS number	Relative abundance (ppb ± SD)^B^	DT in water (ppb)	OAV	Sensory descriptions (Good Scents)^C^
**Esters**								
1147	1141^g^	Isoamyl acetate	130^a^	123-92-2	53,000 ± 9000	2	26,500	Sweet, fruity, banana, solvent
1319	1285^d^	Isoamyl isovalerate	172^a^	659-70-1	40,000 ± 8000	19.9	2,010	Sweet, fruity, Green, Ripe
1213	1181^e^	Isoamyl propionate	144^a^	105-68-0	5000 ± 700	8.6	581	Sweet, fruity, banana
1096	1053^e^	Butyl acetate	116^a^	123-86-4	80 ± 12	66	1	Ethereal, solvent, fruity, banana
1058	1055^g^	Ethyl butyrate	116^a^	105-54-4	50 ± 6	1	50	Fruity, juicy, pineapple,
1300	1263^e^	Isoamyl butyrate	158^a^	106-27-4	33 ± 6	210	0.2	Fruity, green, apricot, pear
1297	1293^g^	Hexyl acetate	144^a^	142-92-7	25 ± 3	2	13	Fruity, green, apple, banana
1033	1012^f^	Isobutyl acetate	116^a^	110-19-0	13 ± 1	66	0.2	Sweet, fruity, ethereal, banana
**Aldehydes**								
1574	1573^a^, 1520^b^	Benzaldehyde	106^a^	100-52-7	600 ± 80	350–3500	2	Almond, fruity, powdery, nutty
2173	2196^g^	Vanillin	152^a^	121-33-5	500 ± 430	20–200	25	Sweet, vanilla, creamy
646	707^a^	Acetaldehyde	44^a^	75-07-0	25 ± 4	15–20	2	Pungent, ethereal, fresh
1109	1083^b^	Hexanal	100^a^	66-25-1	8 ± 1	4.5–5	2	Fresh, green, fatty, aldehydic
1005	988^a^	Pentanal	86^a^	110-62-3	4.20 ± 0.4	1004	4 × 10^–3^	Fermented, bready, fruity
902	884^a^	Butanal	72^a^	123-72-8	1.20 ± 0.2	9–37.3	0.1	Pungent, cocoa, musty, green
**Sulfur compounds**								
1105	1096^a^, 1077^b^	Dimethyl disulfide	94^a^	624-92-0	41 ± 6	12	3.4	Sulfurous, vegetable
637	689^a^	Methanethiol	48^a^	74-93-1	21 ± 4	0.02	1050	Vegetable, oily, alliaceous, eggy
1425	1426^a^, 1430^g^	Dimethyl trisulfide	126^a^	3658–80-8	17 ± 2	0.005–0.01	3,400	Sulfurous, onion, meaty, savoury
680	758^a^	Dimethyl sulfide	62^a^	75-18-3	2.8 ± 0.6	0.3–1	9	Sulfurous, onion, sweet corn
**Furans**								
1258	1241^a^	2-Pentylfuran	138^a^	3777-69-3	12 ± 2	6	2	Fruity, green, earthy, beany
817	799^b^	Furan	68^a^	110-00-9	3.6 ± 0.9	U	U	Ethereal
896	877^a^, 869^b^	2-Methylfuran	82^a^	534-22-5	3.4 ± 0.4	U	U	Ethereal, acetone, chocolate
976	970^c^	2-Ethylfuran	96^a^	3208-16-0	2.0 ± 0.9	U	U	Sweet, burnt, earthy, malty
**Ketones**								
835	819^b^	Acetone	58^a^	67-64-1	190 ± 40	500,000	4 × 10^–4^	Solvent, ethereal, apple, pear
923	910^a^, 907^b^	2-Butanone	72^a^	78-93-3	110 ± 10	50,000	2 × 10^–3^	Ethereal, fruity, camphoreous
1004	980^a^, 979^b^ 973^d^	Diacetyl	86^a^	431-03-8	118 ± 1.6	2.3–6.5	51	Sweet, buttery, creamy, milky
**Alcohols**								
1409	1405^ g^	Cis-3-Hexanol	100^a^	928-96-1	290 ± 50	70	4	Fresh, green, grassy, foliage
1697	1690^ g^	Furfuryl alcohol	98^a^	98-00-0	230 ± 20	U	U	Alcoholic, chemical, musty
**Phenylpropenes**								
2120	2157^d^	Eugenol	167^a^	97-53-0	69 ± 4	6–30	11.5	Sweet, spicy, clove woody
**Monoterpenes**								
1227	1223^g^	d-Limonene	136^a^	5989-27-5	420 ± 60	10	42	Citrus, orange, fresh, sweet

*LRI* linear retention indices, *CAS* chemical abstracts service unique numerical identifier, *m/z* molecular ion peak ^a^mass spectra identified by comparison to NIST database, *DT* detection thresholds from literature (Leffingwell and Associates, 2020), *U*  unknown, *OAV* odour activity value

^A^Literature LRI ^a^Villière et al., (2015), ^b^Thammarat et al., (2018), ^c^Olaoye et al., (2016), ^d^Ricci et al., (2018), ^e^Schubert et al., (2013), Ubeda et al., (2012), ^i^Internal database generated using authentic standards

^B^Abundance values are relative to an internal standard (15 µL 0.1% 3-hepanone)

^C^Sensory descriptions from literature (Good Scents Company, 1980–2020)

**Figure 1:**
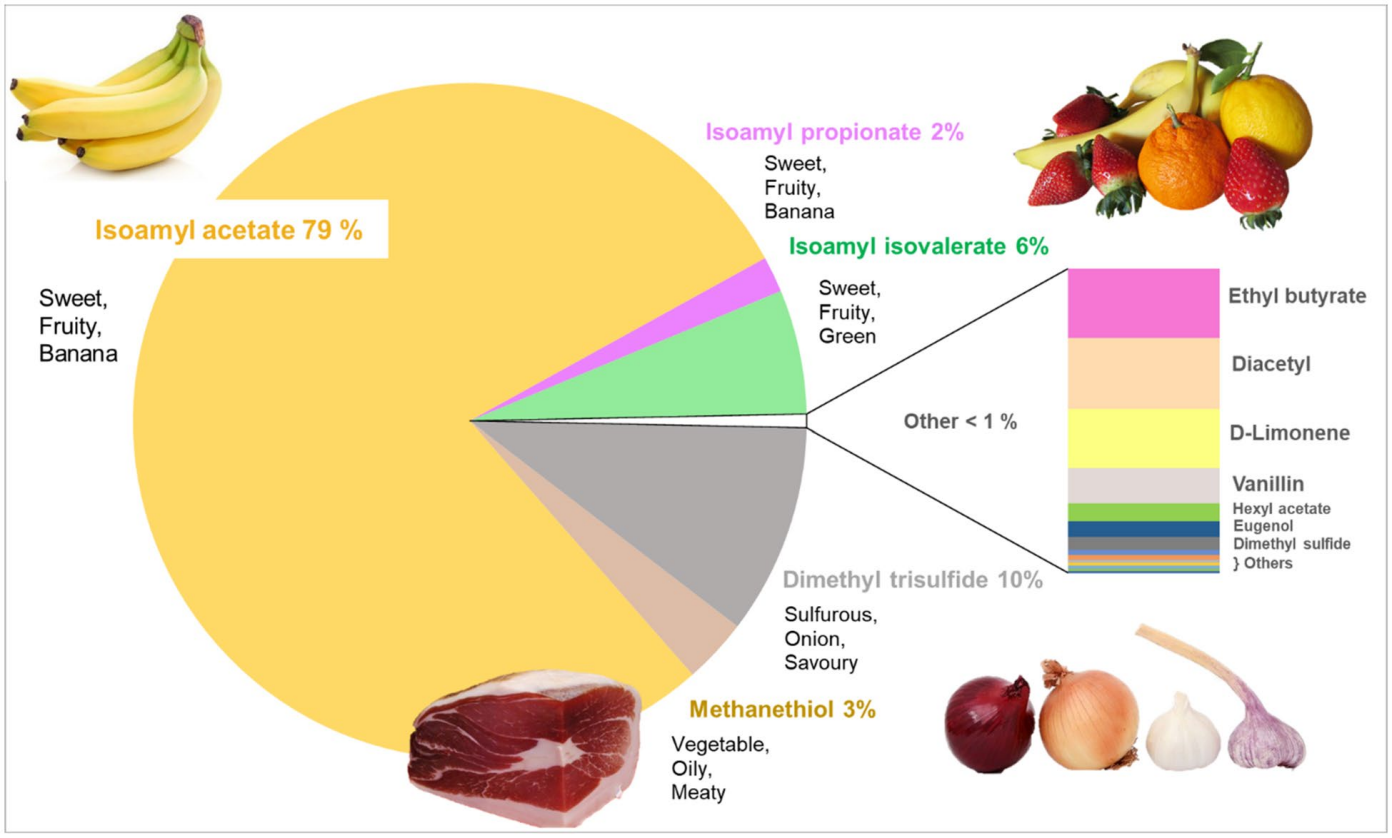
Relative contribution made by volatiles to studied ONS flavour as determined by calculated Odour Activity Values.

Odour Activity Values (OAV) are important when considering the potential contribution of a volatile compound to the aroma or flavour of a food product. OAV can be used to estimate aroma potency in terms of the ratio of the concentration of a volatile to its odour detection threshold^38^ and help to translate the quantitative data gained from GC–MS into sensorial information^39^. As estimated using OAV, Fig. 1 shows the relative contributions (%) made by volatiles to the overall ONS flavour (as a proportion of total OAV), and therefore identifies the volatiles which are potentially aroma active and make the greatest perceptual contributions to the ONS flavour.

OAV require an accurate representation of OT as calculated from the same matrix^38^. The matrix of the studied ONS is a complex aqueous mixture of fat, carbohydrate, protein in addition to micronutrients, so precise OT were not available in the literature for each volatile. Therefore, in line with previous investigations on milk beverages^40^, we used OT values determined in water, to estimate the relative contribution made by volatiles to the flavour and translate the quantitative data into sensorial information^41^. It is important to note that relying on OAV calculations alone could lead to an overestimation or an underestimation of the perceptual importance of these aroma and hence were considered estimates at this stage in our investigations.

#### Gas chromatography-olfactometry-mass spectrometry (GC-O-MS)

GC-O-MS is an alternative approach which combines both the human nose and a mass spectrometer, to confirm which volatiles are aroma active and contribute to the perceived flavour.

**Figure 2:**
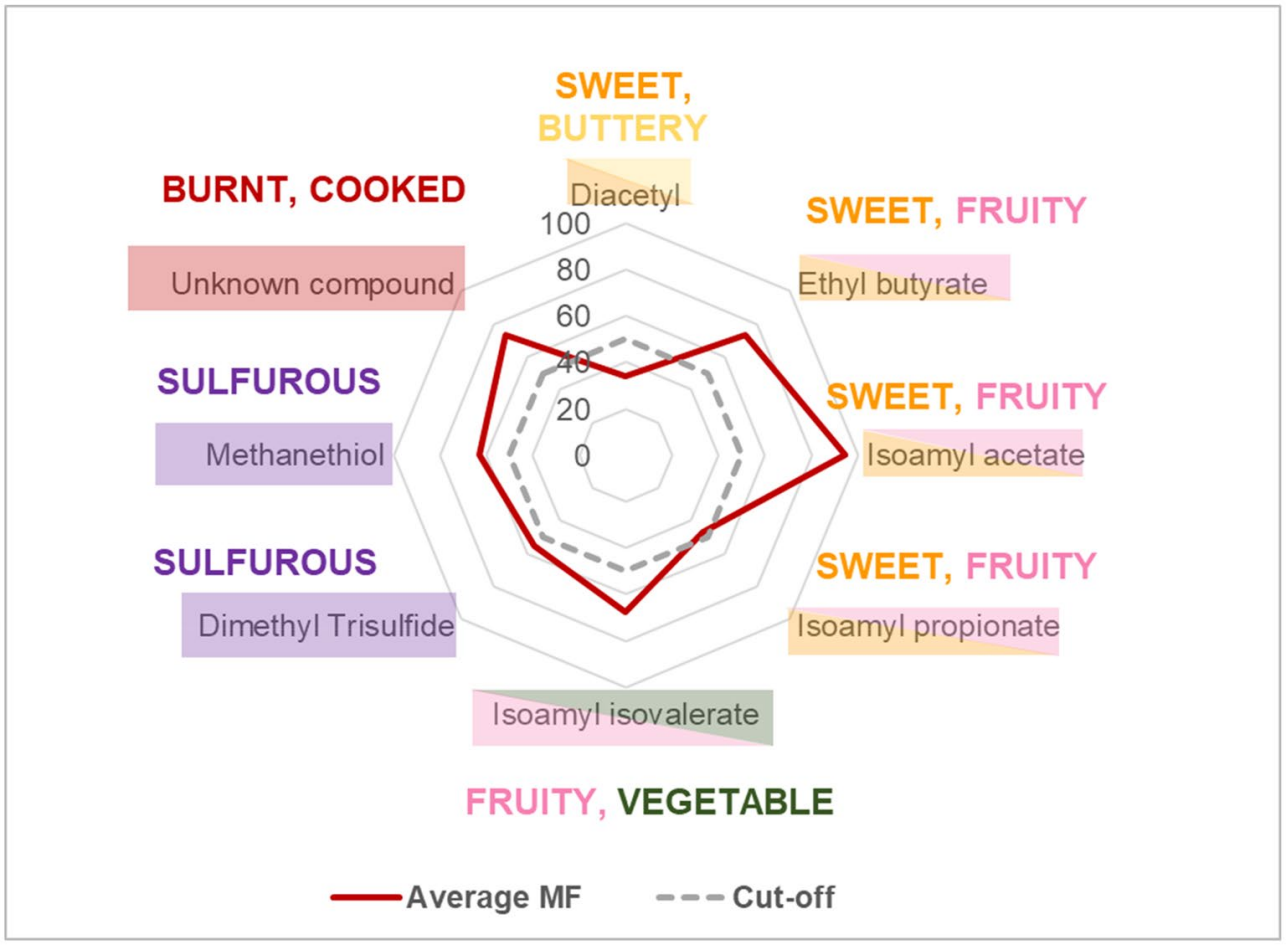
Aroma active compounds detected by all participants (n = 12) during the GC-O-MS analysis. Score shows average MF value for each compound.

Eight aroma active compounds were detected by both the younger and older adults during the GC-O-MS study. These aroma compounds, along with calculated MF scores, are shown in Fig. 2 (data pooled from all participants (n = 12)). In agreement with the GC–MS findings (Fig. 1), these compounds also had the highest calculated OAV, and are therefore confirmed as the volatiles which make the greatest perceptual contribution to the flavour of the studied ONS. The sensory descriptions generated by participants for the detected aroma compounds ranged from sweet and fruity (isoamyl acetate, ethyl butanoate and isoamyl isovalerate) to sulfurous and unpleasant (methanethiol and dimethyl trisulfide) to sweet and buttery (diacetyl) (See Supplementary Table S3 for all descriptions generated).

As measured by GC–MS, fruity esters had a large relative abundance within the ONS (Table 1) and several esters were detected by participants during the GC-O analysis. This is not a surprising finding considering that it is a fruit (banana) flavoured product. For example, isoamyl acetate is an essential flavouring in both natural and artificial banana flavoured foods; in the current study this compound was detected in the highest abundance in the headspace and was detected by all 12 participants (MF% = 94).

A number of volatile compounds were detected, such as sulfur containing compounds (methanethiol and dimethyl trisulfide) and diacetyl, are frequently detected in heat treated milk and dairy products^18–22^. Diacetyl is a rich, buttery, sweet aroma and can form through Maillard reactions and the thermal degradation of sugars^19,20,22^. At a concentration of 38 ppb, it has previously been suggested that diacetyl contributes excessively to the “heated” flavour of Ultra High Temperature (UHT) treated milks^42^. To protect patients who are potentially immunocompromised, and enable long term storage without reliance on refrigeration, many ONS products are sterilised by high temperature treatments during manufacture. It is likely that this heat treatment contributes to the formation of volatiles and may be influential in forming the flavour of the final ONS product.

Volatile sulfur compounds are an important class of aroma compounds, due to their low detection thresholds, strong odour impact and wide distribution in food products, however, they are often overlooked because they are present at trace levels in foods^43^. GC–MS analysis detected four sulfurous compounds in trace concentrations within the headspace of the ONS (Table 1) (methanethiol, dimethyl sulfide, dimethyl disulfide and dimethyl trisulfide). However, during the GC-O-MS study, the only sulfur compounds detected by participants were methanethiol and dimethyl trisulfide; most likely due to their relatively low odour detection thresholds and relatively high estimated OAVs, as shown in Table 1. A frequent source of sulfurous aroma compounds are amino acids with sulfur containing side chains (cysteine and methionine) present in milk proteins, and are liberated during heat treatment (specifically through Strecker degradation)^19,20,22,44^.

Although they can be an essential component of many flavours, sulfurous volatiles can be associated with undesirable off flavours such as cooked and eggy flavours^22^ and concentrations of these sulfurous heat generated compounds have also been found to correlate positively with “heated” flavours of milk products^45^. Vazquez-Landaverde et al.,^40^ quantified sulfur compounds in heat treated milk and proposed that, due to the high calculated OAVs, methanethiol and dimethyl trisulfide are the most important contributors to the undesirable sulfurous note in UHT milk. Considering this previous research, and the relatively high OAV values calculated for methanethiol and dimethyl trisulfide in the current study (OAV = 1,050 and 3,400 respectively (Table 1)). It is likely that these two aroma compounds contribute to the ONS flavour and could possibly be ‘off flavours’ which negatively influence the sensorial experience of ONS. Though, considering that these aroma compounds originate from nutritionally essential amino acids, which cannot simply be removed from a product, it may be more within the interest of manufacturers to prioritise flavour masking, or changes to processing conditions, if aiming to hide or reduce the formation of these high impact aroma compounds.

Despite the “unknown compound” having a high MF% value of 73% during the GC-O-MS analysis, it did not produce a peak on the GC–MS chromatogram, so was challenging to identify. Further investigation, using authentic standards and Linear Retention Indices (LRI), suggested the sulfurous compound “2-acetyl thiazole” (CAS: 24295–03-2, calculated LRI: 1699, literature LRI: 1666^46^) may be responsible for this aroma. When key ions for 2-acetyl thiazole (m/z 43, 58, 85, 99, 112, 127) were searched in the chromatogram they were not present at the specific retention time, and so may not have been detected by the GC–MS. Participants described this aroma as having particularly “Burnt”, “Cooked” and “Nutty” qualities, thus also matching the organoleptic properties of 2-acetyl thiazole^32^.

##### Difference in GC-O-MS perception between older and younger participants

**Figure 3:**
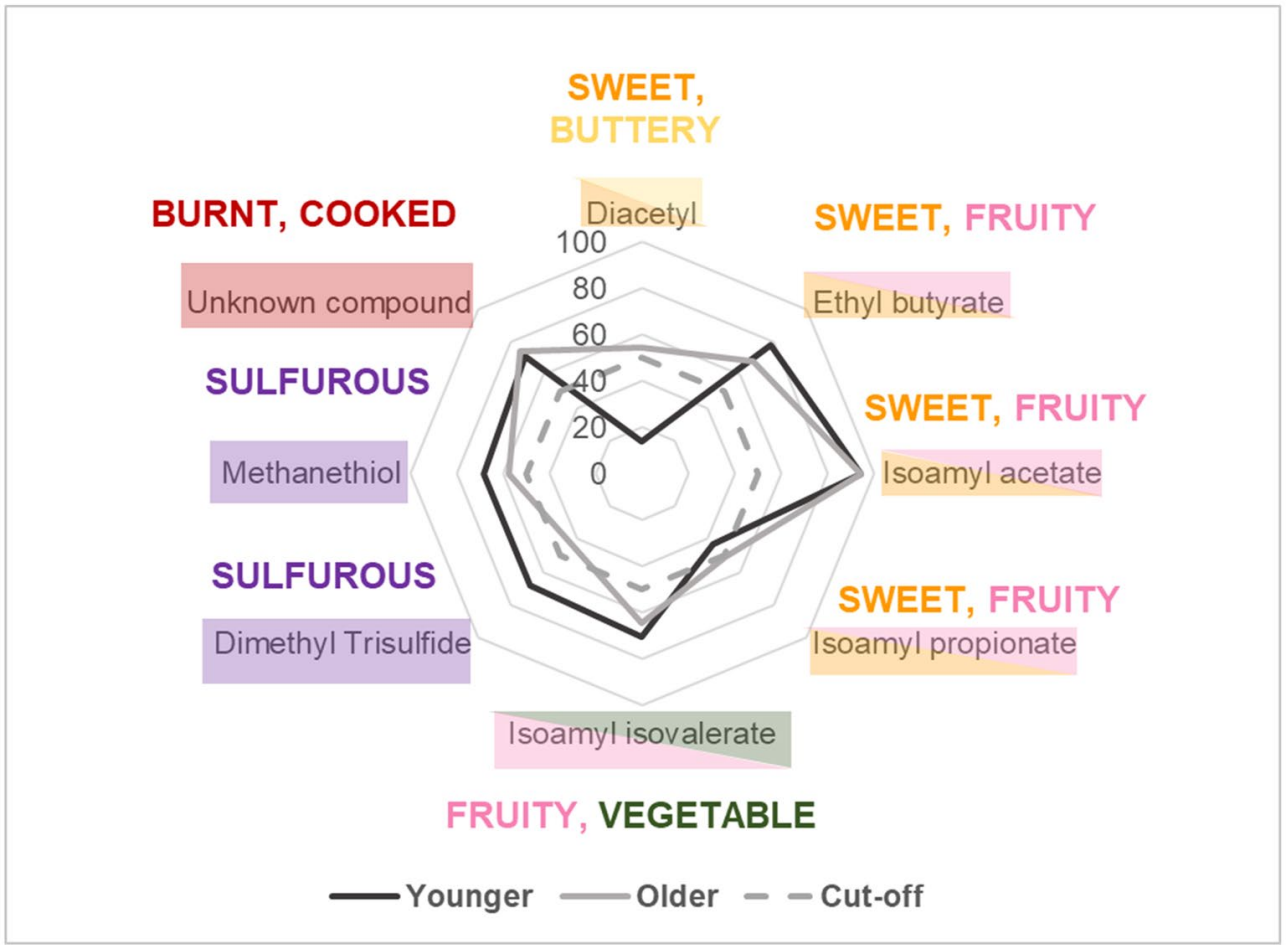
Modified frequency values (%) of younger participants (n = 6, black) and older participants (grey, n = 6). The 50% cut off value is indicated.

Although the same eight aroma compounds were detected by the both age groups, there were some differences between groups, as shown in Fig. 3 (individual and group scores are shown in Supplementary Table S4). The younger adult group had higher MF values for the sulfurous compounds. For example, dimethyl trisulfide reached MF 68% for the younger adult group, whereas the older adult group had a MF value of 43%, which is below the cut off of MF 50%. However, in contrast to what was expected, older adults had higher MF values for the compound Diacetyl; older adults had a MF value of 58%, while younger adults had a much lower MF value of 14%, below the 50% cut off value.

Although recognition thresholds were not directly measured in this study, it was apparent that the ability to recognise and describe an aroma was superior in the younger group. At multiple times during the GC-O-MS study, older adults detected a stimulus but were unable to describe it, so they used terms such as “Indescribable” (ethyl butyrate and methanethiol), and “Indistinct” (isoamyl isovalerate) (see Supplementary Table S3). This suggests that some older adults had more difficulty in recognising and describing the detected stimuli delivered by GC-O-MS. This is supported by other research that found recognition thresholds to be more sensitive to the effects of ageing than detection thresholds^26,47^ and makes reasonable sense considering that the humans’ ability to recognise and describe odours is considered less of an evolutional priority than the ability to detect odours^26,47^.

Humans exhibit considerable variation in the perception of odorants^48^, and due to the relatively small group sizes used in the GC-O-MS analysis, group differences in olfactory ability cannot be explained in depth by the scope of the current method. Factors such as stronger motivation to perform or greater familiarity with the aromas, may have also contributed to differences between age groups. We therefore conducted threshold tests (Experiment 2), with larger group sizes (24 younger adults, and 24 older adults), to better explore differences in perceptual sensitivity between age groups.

### Experiment 2: Age related differences in perception of aroma active aroma compounds

This study used detection threshold tests to investigate the association between ageing and perceptual sensitivity to aroma active compounds. Four aroma compounds were chosen from those detected in the gas chromatography–olfactometry-mass spectrometry analysis (“Gas chromatography-olfactometry-mass spectrometry (GC-O-MS)”) to represent a variety of hedonic, chemical and sensory characteristics. As an additional investigation, the pleasantness of the aroma active compounds, at suprathreshold concentrations, was first assessed by each age group.

#### Aroma compound pleasantness

**Figure 4:**
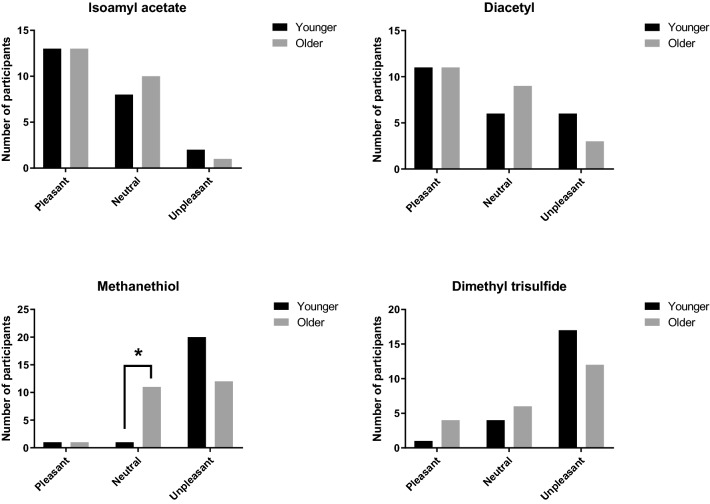
Pleasantness ratings of the aroma compounds, separated by both older and younger adults, for the four aroma compounds studied. Statistically significant differences, as analysed by the Chi-squared test, are marked with * for p < 0.05.

Participants were asked to rate the pleasantness of each aroma compound using a categorical scale: ‘Pleasant’, ‘Neutral’, or ‘Unpleasant’. Isoamyl acetate and diacetyl were categorised as predominantly pleasant compounds or neutral compounds (Fig. 4). Contrary to this, methanethiol and dimethyl trisulfide were rated as predominately unpleasant compounds. A greater proportion of younger adults found the sulfurous aromas to be unpleasant, though, there were not many significant differences in pleasantness ratings between the older and younger groups. The only significant difference found was that significantly more older adults rated methanethiol as a neutral compound (X^2^ = 10.27, df = 2, p = 0.006) compared with younger adults, demonstrating that this compound becomes less unpleasant with older age and similar trends can be observed for the unpleasant sulfurous compound dimethyl trisulfide. These findings are supported by Wysocki and Gilbert^24^ who found that sulfurous mercaptans became less unpleasant with ageing, along with a deterioration of a negative intensity-pleasantness correlation, leading to the interpretation that a reduced perceived intensity of this aroma leads to a reduction in perceived unpleasantness.

It is important to note that, pleasantness of aroma is influenced by concentration^49^; an aroma may be perceived pleasant in low concentrations but unpleasant at high concentrations. This research is the first stage of understanding the hedonic response to aroma active compounds in an ONS; the next stages of research should consider the compounds within a mixture, and a real food matrix, to draw conclusions about the hedonic influence of these compounds on ONS perception.

#### Difference in detection sensitivity between younger and older participants

**Figure 5:**
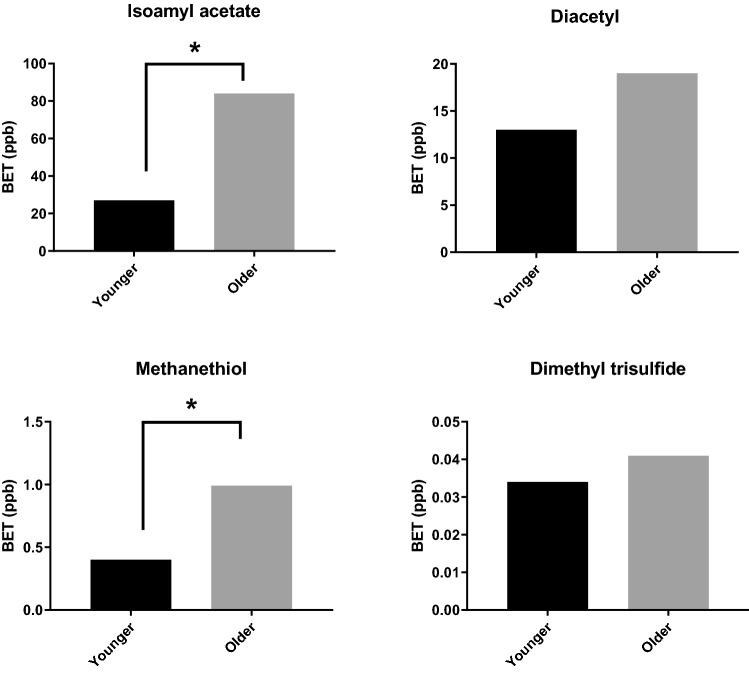
Best estimate detection threshold values (BET), for both younger and older adults, for the four aroma compounds studied. Statistically significant differences, as analysed by The Mann–Whitney U test, are marked by * for p < 0.05.

In line with previous research on age related differences in olfactory sensitivity^24^, older adults had significantly higher detection thresholds for two aroma compounds (isoamyl acetate (p = 0.01) and methanethiol (p = 0.03)) (Fig. 5) demonstrating that older adults need a greater quantity of aroma stimuli in water to detect the aroma.

Age related olfactory impairments have been shown to lead to alterations in dietary habits and food choices^50,51^, poor appetite^52–54^, reduced nutritional intake and status^55^ and play a role in the development or sustainment of undernutrition and frailty^53,54,56^. Across the increasing lifespan, Wysocki and Gilbert (1989) found an almost linear decline in ability to detect isoamyl acetate concurrently with a decline in the willingness to eat a banana flavour food. Considering this previous research, it may be the case that impairments in olfactory sensitivity to identified aroma active compounds alters the perceived flavour or palatability of the studied ONS. Previous research has found differences in flavour preferences for ONS between patients and healthy controls^57–59^. Though, it is important to note that the current study recruited self reportedly healthy older adults as participants. It would be informative to assess this hypothesis with older participants who fall into the ONS consumer group of undernourished older patients, with a greater magnitude of age related diseases and likewise greater medication use. Within this group, age related decreases in olfactory sensitivity may be further impaired.

The cause of this change in olfactory ability is likely to be multifactorial and involve changes in the nose, olfactory epithelium and higher brain regions that receive olfactory input^27^. The complex causes of age related olfactory impairment are discussed in detail in a review by Doty and Kamath^27^ who summarised potential contributing factors to be: altered nasal engorgement and airflow, increased propensity for nasal disease, cumulative damage to the olfactory epithelium from viral and other environmental insults, decrements in mucosal metabolizing enzymes, ossification of cribriform plate foramina, loss of selectivity of receptor cells to odorants and changes in neurotransmitter and neuromodulator systems. It would also not be appropriate to discuss age related olfactory loss without a discussion of the influence of medication within this population. Over 250 medications used to treat age related conditions, such as antihypertensive medications and statins, are known to affect both taste and smell^60^. In the current study, older participants reported taking six times more medications than those in the younger group, including those medications with a recognised effect (a list of the medication classifications that participants were regularly taking are listed in Supplementary Table S2. This is likely to have been a factor contributing to the decreased olfactory acuity of the older group. A comprehensive discussion of the influence of medication and diseases on sensory abilities can be found in Schiffman and Zervakis^60^.

##### Age related impairments are aroma specific

This research has found that age related olfactory loss is not uniform across aroma compounds: detection thresholds were most different between age groups for isoamyl acetate, being over 3 times higher but almost identical for dimethyl trisulfide (See Fig. 5). These findings are supported by previous research. For example, Wysocki and Gilbert^24^ found that age related deficits in olfaction were not uniform across aromas, concentrations or across the life span. In agreement with the current study, they found that reduction in intensity ratings were most pronounced for isoamyl acetate and sulfur containing mercaptans. Interestingly, Seow et al.,^26^ also found that detection thresholds for isoamyl acetate (amongst others) were most significantly impeded by age from the 6^th^ to 8^th^ decade (51–80 years).

The trigeminal sense, responsible for the sensation of tactile, proprioceptive, temperature and painful stimuli^61^ is also vulnerable to age related decreases in sensitivity^62^. Alongside olfaction, many aroma active compounds are also known to stimulate the trigeminal nerve^62,63^ but at a different range of intensities. In particular, diacetyl (sweet, buttery) has been found to modify perception of trigeminal and textural sensations in foods^64^, elicit nasal pungency and can be an irritant at high concentrations^65,66^. Therefore, the extent of the trigeminal component present for each studied aroma active compound may somewhat help explain the aroma specific variation in olfactory sensitivity between the age groups.

This aroma specific loss may also be related to the molecular structure and conformation of olfactory receptors, with some preserved more superiorly across the life course. In line with this theory, Sinding et al.,^25^ found that older adults experience olfactory loss more specifically to heavier molecules. This was not supported by the current study; however, it is likely to be a complex story as humans have ~ 400 different types of olfactory receptor^48^ and volatile aromas have wide ranging physicochemical properties.

Seow et al.,^26^ suggested that unbalanced loss for specific aroma compounds may lead to a distorted perception of aroma mixtures. The current findings are novel because they are the first to apply this theory in real life and focus specifically on aroma specific, age related decline in olfaction to aroma identified within a real product, which can have low adherence in older consumer groups. The new findings from this study generate the new hypothesis that significant age related impairments in the ability to detect pleasant aroma such as isoamyl acetate (sweet, fruity) may negatively alter the perceived flavour of the studied ONS for older adults. Though on the contrary, age related impairments in the ability to detect undesirable sulfurous compounds could be an advantage to the older consumer, because the potentially undesirable off flavour may become less prominent within the overall perceived flavour.

## Conclusions

Oral nutritional supplements (ONS) have the potential to improve the nutritional status of undernourished older individuals however, adequate adherence to the prescribed course of ONS is paramount. Poor palatability, particularly the flavour, has been identified in the literature as an important factor limiting acceptance of ONS. This research is the first to characterise the volatile aroma profile of a commonly prescribed ONS and secondly, to measure age related differences in sensitivity to associated aroma active compounds. Estimated odour activity values (OAV) and gas chromatography olfactometry mass spectrometry (GC-O-MS) identified aroma active compounds in the ONS which were a combination of pleasant fruity esters (isoamyl acetate) and aroma compounds rated as unpleasant (sulfur containing compounds), which are proposed to originate from heat treatment of milk proteins. Older adults had some impairments in their ability to detect aroma compounds at threshold concentrations; in particular these impairments were greatest for the pleasant aroma active compound isoamyl acetate (sweet, fruity) and unpleasant aroma active compound methanethiol (sulfur). We hypothesise that these impairments may alter the perceived flavour profile of ONS for older adults. Future research should consider aroma active compounds in a mixture and the impact of complex food matrices on ONS perception. This is a fundamental study which will aid further research into how the aroma profile, and associated age related impairments in perception, shape the global perception of ONS for nutritionally at risk older individuals. Considering the marked rate at which the population is ageing, and the associated risk of undernutrition, it is vital to understand how to refine flavour formulations for specific age groups.

## Supplementary Information

Supplementary Information.

## Data Availability

The datasets generated and/or analysed during the current study are not publicly available due to commercial confidentiality but are available from the corresponding author on reasonable request.
