# Supplementary material for: Identification of aroma compounds in a commonly prescribed oral nutritional supplement and associated changes in olfactory abilities with human ageing
Source: Sci Rep. 2021 Aug 13;11:16518. doi: 10.1038/s41598-021-95915-6 (PMC8363706; doi:10.1038/s41598-021-95915-6)
Supplement: Supplementary file 1 — Supplementary Information. [file 41598_2021_95915_MOESM1_ESM.pdf]

Identification of aroma-active compounds in a commonly prescribed Oral Nutritional Supplement (ONS) and associated age-related impairments in olfaction

Sophie LESTER[1], Leonardo CORNACCHIA[3], Camille CORBIER[3], Moira A TAYLOR[2], Charfedinne AYED[1], Ni Yang[1], Mui LIM[1], Rob LINFORTH[1], Ian FISK[1*]

[1] University of Nottingham, Division of Food Nutrition and Dietetics, School of Biosciences Nottingham, UK.

[2] University of Nottingham, National Institute for Health Research (NHR) Nottingham Biomedical Research Centre, Division of Physiology, Pharmacology and Neuroscience, School of Life Sciences, Queens Medical Centre, Nottingham, UK.

[3] Danone Nutricia Research, Uppsalalaan 12, 3584 CT Utrecht, The Netherlands

*Ian.fisk@nottingham.ac.uk

Supplementary information.

Supplementary Figure S1: Participant screening and group size

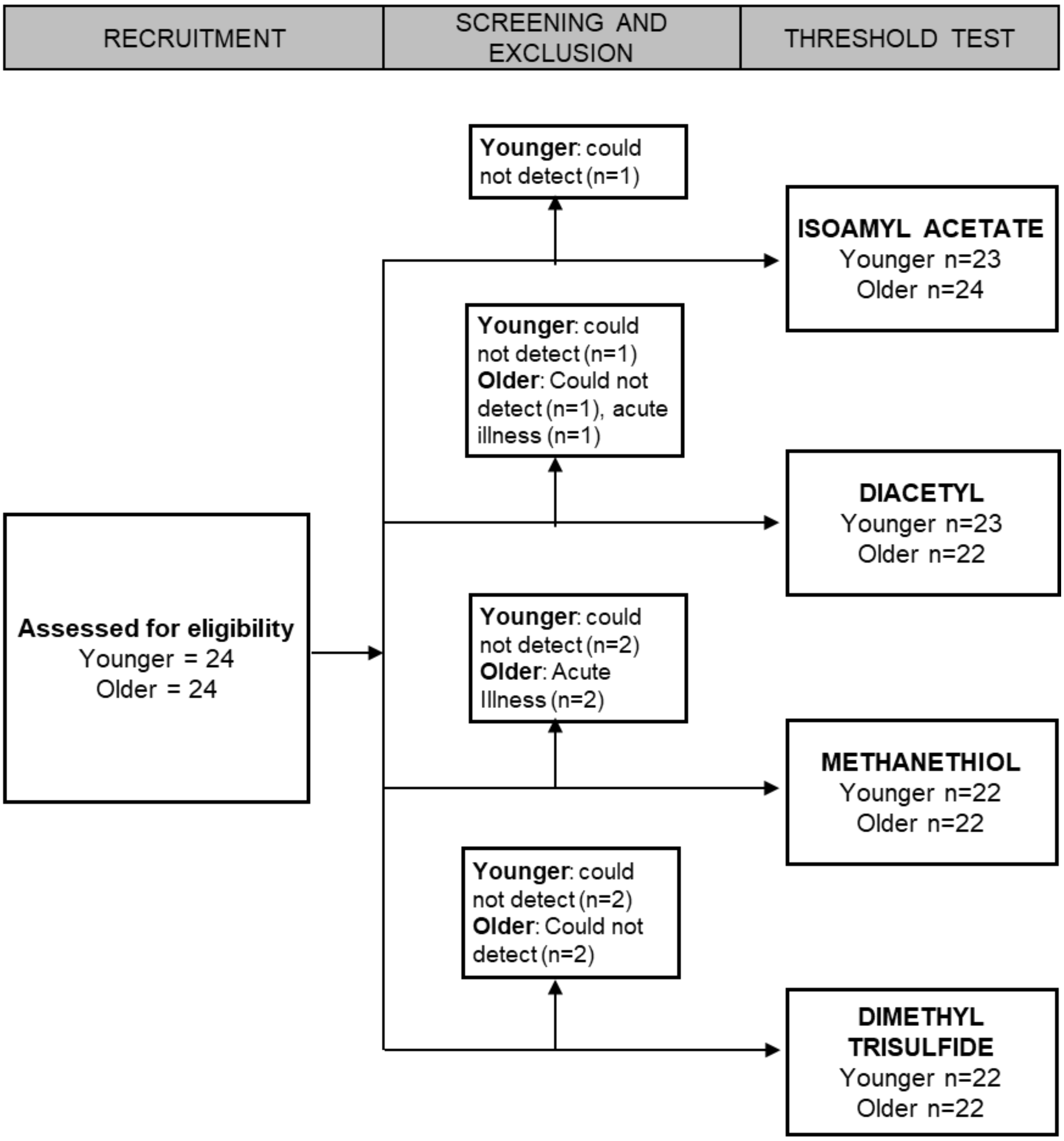

Supplementary Figure S1: Flow diagram illustrating the total number of older and younger participants included in each section of the aroma pleasantness rating and aroma detection threshold test.

Supplementary Table S2: Medication classifications

Supplementary Table S2: Classification of medications regularly taken by participants included in the gas chromatography- olfactometry and detection threshold tests. Numbers in brackets show total amount of participants who reported taking the specific medication class.

Age-group	Medication classification	Mean no. medication per participant
**Gas chromatography-Olfactometry-Mass Spectrometry**		
Younger	None	0
Older	Supplements (6), Calcium Channel Blocker (2), Proton pump inhibitor (2), Alpha blockers (2), 5α-reductase inhibitor (1), Beta-Blocker (1), Xanthine oxidase inhibitor, Angiotensin II receptor blocker (1) Serotonin reuptake inhibitor (1), Statins (1).	3
**Detection threshold tests**		
Younger	Supplements (6), Histamine H1 receptor antagonist (2), Hormonal contraceptive (2).	0.4
Older	Supplements (20), Statins (5), Thyroxine (T4) replacements (3), Proton pump inhibitors (4), Calcium channel blockers (4), Biguanides (2), Angiotensin II receptor antagonists (2), Angiotensin-converting enzyme inhibitors (2), Thiazide diuretics (1), Nonsteroidal anti-inflammatories (1), Serotonin reuptake inhibitors (1), Antimetabolites (1), Anticoagulants (1), Aromatase inhibitors (1), Anticonvulsants (1), Angiotensin receptor blockers (1), Dipeptidyl Peptidase 4 Inhibitors (1), Insulin replacement (1), Hormone replacement (1), Antimuscarinics (1), Bronchodilators (1), Synthetic nucleoside analogues (1), Antibiotics (1).	2.6

Supplementary Table S3: Olfactometry data

Supplementary Table S3: Pooled data showing aroma compounds detected by all participants (n=12) ordered into relative importance by greatest modified frequency (MF %) values. Aroma descriptions were generated by older and younger participants, the number in brackets

Relative impact	Aroma compound name	LRI range	Functional group	Participants aroma descriptions		Pooled DF (%)	Pooled I (%)	Pooled MF% (n=12)
				Older	Younger			
1	Isoamyl acetate	1160-1201	Ester	Banana (3), Sweet (2), Fruity (2), Barley sugar, Unpleasant, Strong.	Banana (3), Fruity (3), Sweet (2), Pear, Estery.	100	89	94
2	Ethyl butyrate	1069-1088	Ester	Sweet (2), Fruity, Indescribable, Strong, Eggy.	Fruity (5), Sweet (3), Estery, Cherry.	92	58	73
3	Unknown compound	1684-1849	Unknown	Cooking (2), Burning, Donuts, Cake, Flour, Unpleasant, Savoury.	Burnt (3), Nutty (2), Bread, Milky, Sweet, Musty, Medicine, Iron, Bitter, Earthy, Dry.	83	64	73
4	Isoamyl isovalerate	1334-1368	Ester	Fruity, Vegetables, Not sweet, Not fruity, Indistinct.	Fruity, Cheesy, Sulfur, Bad, Not pleasant, Off-food, Ammonia-like, Not fruity, Not sweet.	92	50	68
5	Methanethiol	637-663	Sulfur	Indescribable, Unpleasant.	Sulfuric (3), Bad (3), Rotten (2), Faeces, Earth.	75	53	63
6	Dimethyl trisulfide	1431-1457	Sulfur	Sulfur, Bad, Squash, Non-descript.	Sulfur (2), Onion, Cooked, Cabbage, Air-freshener, Chemical.	75	42	56
7	Isoamyl propionate	1233-1240	Ester	Sweet (3), Fruity.	Fruity (3), Estery, Sweet.	67	33	47
8	Diacetyl	1018-1027	Ketone	Sweet (2), Fruity.	Sweet, Caramel.	42	28	34

Supplementary Table S4: Raw Individual and group olfactometry data for each aroma-active compound.

Supplementary Table S4: Detection frequency (DF) and posterior intensity ratings (I) of each participant, for each aroma compound, during the GC-O-MS study. DF of 1 is equal to detection, DF of 0 is equal to no detection. The Sum and percentage (%) for each age-group are shown.

	Aroma compound															
	Isoamyl acetate		Ethyl butyrate		Unknown compound		Isoamyl isovalerate		Methanethiol		Dimethyl trisulfide		Isoamyl propionate		Diacetyl	
	DF	I	DF	I	DF	I	DF	I	DF	I	DF	I	DF	I	DF	I
Younger adults																
Participant 1	1	3	1	2	1	3	1	1	1	3	1	3	1	2	1	2
Participant 2	1	3	1	2	1	2	1	2	1	1	1	1	1	1	0	0
Participant 3	1	2	1	2	1	3	1	2	1	2	1	2	0	0	0	0
Participant 4	1	2	1	1	0	0	1	1	0	0	1	2	0	0	0	0
Participant 5	1	3	1	1	1	1	1	2	1	2	0	0	1	1	0	0
Participant 6	1	3	1	2	1	2	1	1	1	2	1	2	1	2	0	0
Sum	6	16	6	11	5	11	6	9	5	10	5	10	4	6	1	2
%	100	89	100	61	83	61	100	50	83	56	83	56	67	33	17	11
Older adults																
Participant 7	1	3	0	0	1	2	1	1	0	0	0	0	0	0	1	2
Participant 8	1	3	1	3	1	3	1	3	0	0	1	1	1	2	0	0
Participant 9	1	2	1	1	1	2	1	1	1	1	0	0	0	0	0	0
Participant 10	1	3	1	3	1	3	1	2	1	3	1	1	1	2	1	1
Participant 11	1	2	1	2	0	0	0	0	1	2	1	1	1	2	1	2
Participant 12	1	3	1	1	1	2	1	2	1	3	1	2	1	1	1	3
Sum	6	16	5	10	5	12	5	9	4	9	4	5	4	7	4	8
%	100	89	83	56	83	67	83	50	67	50	67	28	67	39	67	44
